# Institutional Factors Associated with Equitable Cancer Care Provision for Culturally and Linguistically Diverse Populations in Queensland, Australia: A Critical Race Theory Analysis

**DOI:** 10.1007/s40615-025-02477-8

**Published:** 2025-05-16

**Authors:** Brighid Scanlon, Natasha Roberts, David Wyld, Ghasem Sam Toloo, Jo Durham

**Affiliations:** 1 Faculty of Health, Queensland University of Technology, Kelvin Grove, Brisbane, QLD Australia; 2 Royal Brisbane and Women’s Hospital, Cancer Care Services, Herston, Australia; 3 Faculty of Medicine, University of Queensland, Brisbane, Australia; 4 Surgical, Treatment and Rehabilitation Service, Herston, Australia

**Keywords:** Health equity, Cancer care, Health services, Racialisation, Service delivery, Universal health coverage, Medical oncology, Institutional racism, Critical Race Theory

## Abstract

**Background:**

Cancer inequities for Culturally and Linguistically Diverse (CALD) populations have been demonstrated in Australia. Historically, research has focused on individual factors, but addressing structural and institutional determinants is crucial for equitable care provision. This study utilised Critical Race Theory to examine institutional factors impacting equitable care provision.

**Methods:**

We undertook a qualitative exploration of a large tertiary hospital in metropolitan Queensland. Institutional barriers, facilitators, and staff experiences regarding equitable care were explored through semi-structured interviews (*n* = 21). Participants included oncology registered nurses (*n* = 6), oncology doctors (*n* = 5), specialist nurses (*n* = 7), and executive-level staff (*n* = 3). Data were analysed using The Framework Method.

**Results:**

Findings revealed an inflexible health system with strong deficit framing of CALD patients. A reliance on assumptions and informal mechanisms to address the needs of CALD patients resulted in suboptimal practises such as simplified information sharing, use of unqualified interpreters, limited treatment access, and avoidance of psycho-social discussions. Staff reported experiencing moral conflict when providing care discordant with their professional values.

**Conclusions:**

This study demonstrates the need for cultural and structural reform within Australian health services. Adapting services to promote equity will have demonstrable benefits for patient outcomes, quality of care, and staff wellbeing.

## Background

Australian healthcare institutions are inextricably linked to, and continue to be influenced by, Australia’s colonial history and the legacy of the 1901–1973 *White Australia Policy* [1]. This discriminatory policy explicitly restricted the levels of “non-white” migration to what is now known as Australia [2]. Fifty years on from the abolition of this policy, the ramifications of this divisive ideology remain visible throughout Australia, manifesting as widespread socio-economic and health inequities for “non-white” Australians, such as Aboriginal and Torres Strait Islander peoples and racialised migrant populations [1, 3, 4]. Critics argue that this policy directly enabled the institutionalisation of racism and the normalisation of “whiteness”, whereby white identities are centred to the exclusion or detriment of racialised populations throughout Australia [1]. Indeed, in 1901, then Prime Minister Alfred Deakin positioned cultural and linguistic diversity as a threat to national unity when he stated: “unity of Australia is nothing if it does not imply a united race…Other races are to be excluded by legislation if they are tinted to any degree. The yellow, the brown, and the copper coloured are to be forbidden to land anywhere” [1]. This quote demonstrates that cultural, racial, and linguistic homogeneity were not merely goals of Australian society but were explicitly embedded within legislation and government policies, to varying extents, until 1973. Due to this historical and political context, it is unsurprising that Australian health institutions continue to foster and perpetuate structural health inequities amongst those with racialised identities [1, 5]. Contemporary institutionalised racism refers to the systemic practises, policies, and cultural norms within institutions which perpetuate inequities [6]. In healthcare, this has been characterised as white-centring health settings, which treat whiteness as the “norm” from which all others deviate, through “colour-blind” medical practises, and through uncritical research and health policies that perpetuate community deficit narratives [3, 7, 8]. Ultimately, institutional racism sustains entrenched health inequities, such as a disproportionate burden of disease and lower survival rates for racialised populations, at both the individual and community levels [9].

There is strong international evidence highlighting inequities for racialised populations, particularly in complex chronic diseases, such as cancer [10–12]. In Australia, inequities have been established for Culturally and Linguistically Diverse (CALD) populations in healthcare access, outcomes, and treatment quality [10, 13]. In the field of cancer care, CALD populations experience inequitable access to screening services [14, 15], have poorer quality of life [16], lower clinical trial participation [17], poorer communication with clinicians [5], and challenges with health system navigation [18]. Despite these established disparities, Australian research to date has focused heavily on individualistic, behavioural explanations for disparities, seldom exploring the historical, social, or institutional drivers of health inequities [19, 20]. This gap is notable, as health institutions are increasingly recognised as both contributors to health inequities and essential resources for promoting equitable care [21].

Australia, like most Organisation for Economic Co-operation and Development (OECD) nations, has adopted a system of Universal Health Coverage (UHC), which was defined by the 2005 World Health Assembly as “access to key promotive, preventative, curative and rehabilitative health interventions for all at an affordable cost, thereby achieving equity in access” [22]. This has led to significant gains in overall population health; however, failures to make equity an explicit priority in UHC policies have resulted in persistent inequities amongst marginalised and racialised populations [22–24]. In response to this, health equity has been increasingly prioritised within global health systems, policies, and research [25–27], creating an urgent need to understand how health institutions committed to UHC integrate and promote equity. This study utilises Critical Race Theory as a tool to explore the structural origins of persistent cancer-related inequities for CALD populations in Queensland, Australia.

## Methods

### Theoretical Approach

Critical Race Theory (CRT) has been increasingly utilised in population health research, as a way to disrupt the dominant, white-centering narratives through recognising the historical and contextual factors that influence health inequities, such as colonialism, racialisation, and institutional racism [28]. Racialisation refers to the social construction of racial categories, which leads to the unequal distribution of power and differing social, economic, and health outcomes [29]. Critical race theorists argue that racialisation is an active and ongoing process, whereby a dominant group subordinates another, often based on physical characteristics [30]. The legacies of colonialism and racialisation persist in the structures of institutions and in the power these institutions exercise over individuals [31]. Structural and institutional racism refer to the macro-level systems within institutions that reproduce the oppression of racialised populations, whilst normalising white identities [31]. Institutional racism is now accepted as a key health determinant; however, research has been slow to identify and investigate the mechanisms thorough which it engenders health inequities [32]. In this study, CRT was employed to shift the focus from individual-level factors and towards the structural and institutional factors associated with equitable care provision [33].

### Researcher Positionality and Reflexivity

Positionality encompasses the wider social, political, economic, religious, and intellectual contexts that influence interpersonal relationships and the research process [34]. Critical reflexivity was essential for addressing the inherent power differentials, potential biases, and privileged position of the lead researcher, who is a non-CALD identifying clinician. Reflexivity involves maintaining ongoing, subjective self-awareness that serves as a tool for critically examining one’s perceptions [35]. To support this process, the lead researcher maintained a reflexive journal that critically examined her thoughts, experiences, and pre-existing biases and assumptions that could influence interactions with participants and the interpretation of their responses [36].

### Setting, Participants, and Consent

This study utilised semi-structured interviews with hospital staff. The interview guide was informed by the key tenets of CRT, namely racialisation, race consciousness, social location, and action, and reflected on the findings of previous research conducted by the research team [8, 10, 37, 38]. The interview questions (Supplementary Material 1) were exploratory in nature and aimed to understand perceived barriers, facilitators, and current strategies that influence the provision of equitable cancer care.

Participants were recruited via a service-wide email advertisement regarding the study. Participants were also informed of the interviews via word of mouth or recommendation from a previous participant. Participants included a cross-section of clinical, management, and executive-level staff working in a major, tertiary hospital in metropolitan Brisbane, Queensland. All participants, excluding executive-level staff worked directly within cancer care services. The lead researcher (BS) conducted (*n* = 21) interviews, continuing until data saturation was reached. This was determined by the absence of new themes during the review of consecutive interviews and consensus agreement between the two researchers (BS and NR) [39]. Inclusion criteria for participation were as follows: (1) Currently employed by the relevant hospital and health service, (2) A member of front-line clinical staff working in cancer care services, or a mid-senior management or senior executive-level staff member, with knowledge of cancer care services, (3) Aged over 18 years of age. Exclusion criteria were as follows: (1) Patients or their family members, (2) Those aged under 18 years. All participants were given a detailed written information sheet prior to the written consent process. Participant consent was guided by Chapter 2 of the National Statement on Ethical Conduct in Human Research 2007 (updated 2018) [40]. Verbal consent was obtained and recorded as part of the interview process. Consent was also reaffirmed throughout the interviews and at the conclusion of each interview.

### Data Collection and Analysis

Interviews were recorded using the videoconference software Microsoft Teams [41]. The video files were destroyed immediately, and the audio data were transcribed verbatim. Once the transcripts were checked by the lead researcher for completeness, the audio files were destroyed. The interview transcripts were stored in a password protected server and used for the analysis. Data were analysed using The Framework Method, developed by Ritchie and Spencer [42]. The lead researcher (BS) and a secondary researcher (NR) then underwent data familiarisation through separately reading interview transcripts and contextual notes taken during interviews [42, 43]. Key substantive lines, phrases, and values were then coded manually and independently. The two researchers (BS and NR) then discussed their codes and formed an analytical framework. The data were then summarised into a matrix and organised by categories, displayed in Table 1 [42, 43]. An inductive approach to qualitative analysis was utilised to allow for unexpected findings and different perspectives from the two researchers [43, 44].

**Table 1:** Sample of the analytical matrix

	Environment	Culture	Communication	Structure
Interview 1	“… fast-paced environment…you don’t get a chance to develop that rapport” “A quiet place to actually build rapport”	“It’s very clinician dependent” “You can’t just make assumptions” “it’s a bit difficult… he doesn’t speak English”	[access to interpreters] “I have used Google translate…when the translator has called in sick” “…there can be that power imbalance… prevents that open communication”	“…I find I have to overstep my boundary in my position to be able to provide …additional care” “You book an interpreter for that whole time, which you get questions about…they’re very expensive”
Interview 2	“… I think as soon as you add another layer to into a stretched system, people just go, oh well, I won’t tell them” “… it gets deprioritised for me”	“…I have made some assumptions in the past” “…I’ll ask a lot more questions versus somebody else who may not look like they’re culturally diverse”	“I try to give written supplementation… I don’t know that I use interpreters well” “I have moved to simplifying my language… because I worry about what’s getting translated”	“… I’m not sure I’m asking the right questions” “… this puts me in a very difficult position”
Interview 3	“…quiet spaces…even sharing a two bedded bay…having another patient and everyone hearing” “…not having the time to really explore that particular patients’ needs”	“…mainstream sort of white, Caucasian lifestyle, privileges…people do not just live in this bubble… there’s this whole world outside your bubble and you have to be sensitive and aware…”	“Communication is the biggest barrier…getting interpreters can wait if it’s not a life and death situation” “…having easier access to interpreters…having written resources to give people”	“Just allow you to be a little more prepared rather than the patient just turning up and having to troubleshoot and try to manage on the spot” “…often you have to unfortunately just get by…”
Interview 4	[reduced information sharing] “…they’re not getting the same degree of information and support as an Australian-born-type person because of language barriers and the lack of information in their own language” “…I don’t think we give as good service to those from diverse backgrounds, simple because the resources aren’t there”	“…if they have poor English, you know that straight away…if they’re from a different culture…over time…you should find out along the way” “…skin colour can indicate they are not Caucasian…but then it doesn’t mean that they are diverse either because they might be highly Westernised”	[using multilingual staff] “…having that person as a support was really, really good for her” [access to interpreters] “…having someone more accessible… but if not…then they need to have someone like a family member that can speak English…”	“…looking online I couldn’t find a lot of information…I’ve got no idea what they are and I couldn’t find much” “…something that’s concerned me a lot is that we have so few resources available in other languages…and we’ve got so many people from different backgrounds…and you look around and all the information is in English”
Interview 5	“…we don’t have any written information to give people in different languages…” “You know, if time was no problem then obviously you’d go through all the checks…and make sure they understand…” “…in those situations, you’re just trying to communicate the basics…we miss out on quite a lot…the psychological side…there’s just no time to go through all of it”	[assumptions] “it’s very much on your own assumptions…I guess there’s a clue in peoples’ names…you can never make that assumption…that’s not really good enough” “I am diverse, but I don’t have any problems accessing healthcare and so I consider myself on an equal level to those born in Australia…it’s usually people who are suffering as a result of that diversity…”	[family as translators] “…I have concerns that sometimes when family are used to translate …that the whole picture is not there… I also have concerns about people who appear to have a proficient understanding of English…put them in a hospital environment, put them under stress, tell them they’ve got cancer…their level of proficiency is gonna go down”	“I don’t feel that they’re well set up for supporting people in that situation…your stress levels are high, and it would be great if we could put things in place in the future to improve those outcomes…but also improve the workload of everyone in the hospital…it’s not right…we haven’t got it right”

### Development of the Conceptual Framework

**Fig. 1:**
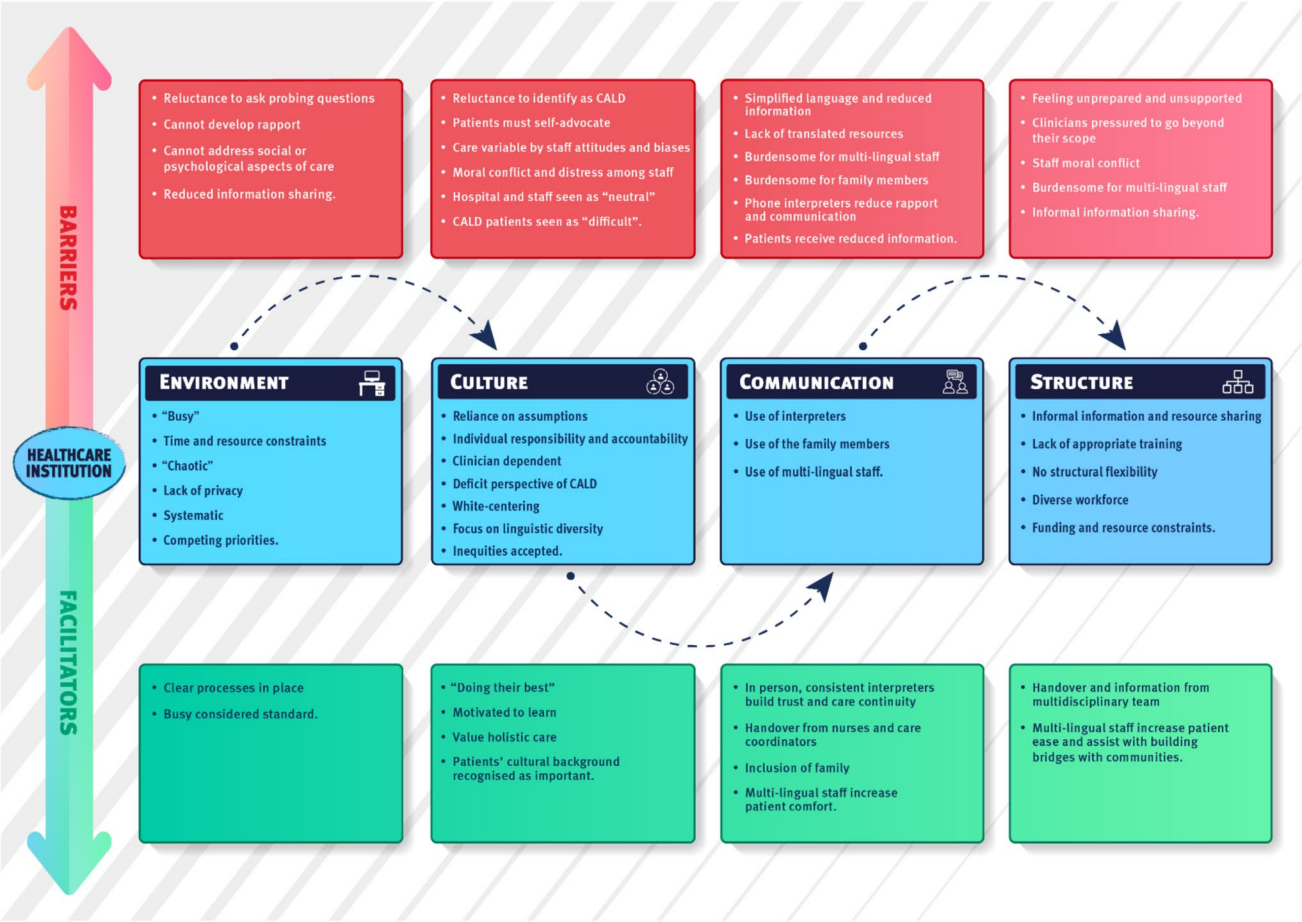
Conceptual framework

A conceptual framework was developed using the key themes and concepts identified in the interviews, displayed in Fig. 1. Two coders (BS and NR) reviewed the dataset to assess the extent to which the framework reflected the institutional barriers and facilitators identified by the participants [45, 46]. The framework was developed and discussed by the two data coders and then reviewed by the wider authorship team until agreement was reached.

### Ethics

Prior to commencing this study, ethical approval was sought and obtained through the Gold Coast Hospital and Health Service Ethics Committee: (HREC/2022/QGC/88330). All identifying information was removed prior to data analysis, and participants remained confidential throughout the analysis process. All participants were offered an honorarium café voucher for their participation, and mental health supports were available for participants, as part of their Employee Assistance Service, which aims to support staff with challenges associated with their workplace [47].

## Results

There were 21 (*n* = 21) key informants who individually participated in an in-depth, semi-structured interview. Each interview ranged from 35 to 70 min in duration. Of these, six (*n* = 6) were oncology registered nurses, five (*n* = 5) oncology doctors, seven (*n* = 7) specialist nurses, and three (*n* = 3) executive level hospital staff. Of those interviewed, seventeen (81%) were female and four (19%) were male. Six (29%) of the participants identified as CALD, thirteen (62%) did not identify as CALD, and two (9%) were unsure, by their own definition of CALD. Most participants (91%) had greater than 5 years’ experience in their role. Participant characteristics are displayed in Table 2. Common themes were identified regarding the institutional barriers and facilitators for care provision. We identified four key institutional categories (environment, culture, communication, and structure) which were used as the analytical framework. The resulting conceptual framework is depicted in Fig. 1. Many of the factors identified could be considered either facilitators or barriers, depending on the participants’ experiences and perspectives.

**Table 2:** Participant characteristics

Characteristics	*n* (%)
Profession	
Oncology Registered Nurse	6 (29%)
Oncology Doctor	5 (24%)
Specialist Nurse	7 (33%)
Hospital Executive	3 (14%)
Total	21 (100%)
CALD identifying	
CALD	6 (29%)
Not CALD	13 (62%)
Unsure	2 (9%)
Total	21 (100%)
Sex	
Male	4 (19%)
Female	17 (81%)
Total	21 (100%)
Years of experience in role	
< 5 years	2 (9%)
> 5 years	19 (91%)
Total	21 (100%)

## Environment

### Competing Priorities

The institutional environment was consistently described as busy, chaotic, process driven, and lacking appropriate time and resources for quality, culturally safe care. This led to a reluctance amongst staff to ask deep or probing questions, particularly of CALD patients, for fear of uncovering an issue that time did not permit them to address.

And of course, you don’t want to open a can of worms… you’re kind of praying that they’re going to say that everything is fine because…there’s not enough time to sort things out… I think as doctors we’re quite guilty of not digging deep in many situations…psychosocial being one of the worst but also sexual health and mental health… you just don’t have enough time… and you hope that there will be someone else… who might talk to them about these things, but no, probably not.

Interview 5 (Doctor)

This participant reflected that the busy environment reduced the depth of their communication with patients, particularly CALD patients.

In those situations where you’re just trying to communicate the basics. Yeah… we miss out on quite a lot...the psychosocial side. I think we miss that entirely in the CALD population, because there just isn’t time…

Interview 5 (Doctor)

Several participants reported that the busy environment reduced their ability to develop rapport and communicate with CALD patients, but privacy was identified as an environmental facilitator.

A quiet place to actually… build that rapport can be difficult at times… I’m lucky that we have our assessment room when it’s available… you can actually close the door... It’s a bit uncomfortable in the clinic, in the waiting room when it’s incredibly busy

Interview 1 (Specialist Nurse)

There were very few additional facilitators identified regarding the current institutional environment. Those identified included having clear processes in place, and one clinician expressed that being busy was a part of standard practise and therefore should not be considered a barrier to care provision.

I really try not to look at it as though we are so busy, this is our job. This is our core business… were going to be busy always and that’s just our standard.

Interview 18 (Specialist Nurse)

## Culture

### Understanding Assumptions

The concept of assumptions was consistently referred to throughout the interviews. Participants described being both avoidant of and reliant on assumptions. Staff described not wanting to make assumptions about patients regarding their cultural and linguistic backgrounds or their values and preferences.

I don’t want to judge, and I don’t want to make an assumption…I don’t want to make an assumption at all.

Interview 18 (Specialist Nurse)

However, it was also clear that clinicians relied heavily on unspoken assumptions to identify and care for CALD patients. Participants reported that they had no formal mechanism to identify CALD patients and instead relied on factors such as patient names, skin pigmentation, accents, and mannerisms. Language was the most consistent characteristic used to identify CALD patients.

Language for some, in some respects their appearance, you know, skin colour can indicate they are not Caucasian…but that doesn’t mean that they are diverse either because they might be highly Westernised…

Interview 4 (Registered Nurse)

I guess there’s a clue in peoples’ names, but you can never make that assumption… that’s not really good enough. I feel we need a good way of doing that.

Interview 5 (Doctor)

Assumptions were also central to identifying patient needs and assessing if their needs were being met.

…I don’t actually have a mechanism to make sure that they’re getting the right information. I just assume that because they’re taking it [medication] and coming, that all is well”

Interview 2 (Doctor)

This reliance on assumptions was identified as a significant barrier to care provision, as it reinforced that CALD patients and their needs are largely invisible within the health service.

You can’t support something that you don’t see, and you can’t support a population that is invisible

Interview 13 (Executive staff)

### Taking Responsibility and Accountability

Participants described a culture of individual responsibility within the institution, with family members often utilised when the patient is unable to advocate for their own needs.

they [family] are often the ones relaying messages or helping us understand if we’re meeting their needs…I don’t think we’re measuring…in any way, how successful we are in achieving those needs for people. And often it’s probably the bare minimum.

Interview 6 (Registered Nurse)

Care was often described as “clinician dependent”, with many participants acknowledging the influence of individual staff attitudes and practises on the provision of equitable care.

Some clinicians are better than others at understanding…I’ve witnessed it firsthand, there’s a huge discrepancy in appreciation of different backgrounds and how they can affect care…some clinicians will tend not to…explore those issues… and some will disregard them…

Interview 14 (Doctor)

Participants highlighted the risks of care variation depending on individual staff attitudes, which can limit the ability of individuals with limited English proficiency (LEP) to self-advocate when faced with substandard or inconsistent care.

… a system, particularly a public system, should not be reliant on the attitudes and behaviours of people. You know whether they are champions for equity or not. It shouldn’t be reliant on people constantly putting their necks out or advocating for change”

Interview 13 (Executive)

The risk to those with LEP was expanded on by another participant.

Unfortunately, it varies on health professionals…and it’s not surprising when people can’t hold us accountable, then they do fall, I guess out of the priority list.

Interview 6 (Registered Nurse)

### White-Centering and Deficit Framing of CALD

Two clinician participants reflected on the role of the health system in marginalising CALD populations and their own role in privileging the “mainstream” or majority perspective. They expressed frustration with the current system and discomfort regarding their position within it.

What’s happening to people who are… in the margins? because they don’t fit into this white, English-speaking world, western world…and if you’re outside of that, you’re identified as ‘different’.

Interview 7 (Registered Nurse)

…they don’t have a voice and we are just fixated on whether people are attending their appointments and putting people in boxes [like did not attend] that we are not always looking at the patient.

Interview 19 (Specialist Nurse)

Despite conveying insights into the marginalisation of CALD populations within healthcare institutions, there was a clear distinction between “us” and “them” when participants were discussing patients and staff.

You’ve got that overarching idea without even thinking because you know, you live in the mainstream and then you just take all those stereotypes, you take on this patriarchal thing…where you’re the dominant and everyone else is down there…but you don’t even realise you’re doing that, and it’s not actually your intention…you don’t want to do that.

Interview 7 (Registered Nurse)

Conversely, one non-clinician participant reflected that CALD populations needed to change and adapt to fit the health system.

we see with culturally and linguistically diverse people… will say… you know, ‘at home I used to do this and this’…You know, this is your home…And you’ve got to concentrate on building this to be your home.

Interview 8 (Executive)

When asked about the needs of CALD patients within the health service, the majority of participants responded with strong associations with the concepts of “vulnerability” and “suffering”. The definition and identification of CALD patients was often used as a proxy to identify social or structural vulnerability, with several staff reporting a need to identify those vulnerable to ill health or those “suffering as a result of their diversity”.

It’s about barriers and recognising vulnerabilities and not, unfortunately so much about who that person is…which I do feel quite disappointed about when I think about it that way, because…that’s probably a large part of their identity.

Interview 6 (Registered Nurse)

This essentialist, deficit perspective of the CALD identify was further reinforced by some participants acknowledging that they could be considered CALD, but they choose not to identify as such because they do not see themselves disadvantaged, or vulnerable to poorer health outcomes. This reinforces that the strong “othering” discourse used to distinguish staff and CALD patients persists, even in the language employed by CALD staff members.

I am diverse, but I don’t have any problems accessing healthcare, so I consider myself on an equal level to those born in Australia…so I guess what’s important in terms of healthcare, it’s usually people who are suffering as a result of that diversity. Whether they feel persecuted or there’s a lack of access for that reason or a lack of information for that reason.

Interview 5 (Doctor)

### Staff Awareness of Inequities

Participants were asked if they found it surprising that CALD populations were experiencing inequitable cancer treatment outcomes within the health institution they worked in, and only one participant said “no”. Overall, there was an acceptance that inequities were an unfortunate reflection of the current system.

Unfortunately, they’re not surprising to me, which I feel, to be honest, quite sad about…because we’re not doing the things…that would promote quality care…and informed decision making and patient-centered care. So really its zero surprise to me that if we’re not doing those basic things…their care is suboptimal.

Interview 6 (Registered Nurse)

It doesn’t [surprise me] because I’ve heard it before. But no, it disappoints me.

Interview 5 (Doctor)

### Personal Values

Aspects of the institutional culture that were considered facilitators for equitable care provision were the attitudes and values of staff members. Staff frequently reiterated that they felt they were “doing their best” with the resources they had available. Staff felt that the majority of people working in the institution were well intentioned. Most participants recognised the importance of patients’ cultural and linguistic background and valued being able to provide holistic, tailored care to patients. The majority of participants were reflective of their own personal background and open to continued education and training regarding the provision of equitable care. Staff described experiencing moral conflict when providing care that does not align with their personal and professional values but reflected that they felt they had no power to change the process.

You feel as though you might be compromising your skill set and values as a nurse when you’re not free to provide that holistic care…

Interview 12 (Registered Nurse)

## Communication

### The Role of Interpreters

Language was the most consistently identified barrier to communication with CALD patients, with the use of interpreters reported as a key aspect of quality care provision. Access to consistent, in-person interpreters was frequently highlighted as a facilitator; however, it was acknowledged that this was uncommon. Clinicians highlighted the time pressures associated with interpreter usage that are not compensated for in appointment times.

we get…allocated a 15-minute slot…time is a big issue…there doesn’t seem to be much, you know, in the system, there doesn’t seem to be much appreciation in terms of…changing clinic times to reflect that…

Interview 14 (Doctor)

This led to many staff members, particularly doctors, to alter their communication, through simplifying language and limiting the depth of information that they conveyed via an interpreter. It was acknowledged that this did lead to some patients receiving more superficial and lower quality information; however, it was seen as their only option within the time and resource constraints.

I have moved to simplifying my language with an interpreter because I worry about what’s getting translated…Yeah…is that fair that they don’t get the same level? Probably not. But if they get nothing across, that’s even worse.

Interview 2 (Doctor)

It is very challenging [communicating] through an interpreter because it’s a complex decision and you know; consent says people have to understand information… and that takes so much longer…I do tend to simplify things just to get the main points across. But yes, I think that’s an issue…

Interview 14 (Doctor)

One doctor, who identified as CALD, reflected on this as a significant issue, as they noted that this practise reinforces an assumption that LEP equates to poor health literacy. However, they stressed that the simplification of information was for the ease and benefit of the interpreter, not the patient.

You’re simplifying language, not necessarily for the patient, because I guess that assumes that the patients’ health literacy isn’t that good, just because their English might not be as good, but actually their health literacy might be excellent... In their own language.

Interview 15 (Doctor)

Lack of access to timely interpreter and translation services was also linked to a lower enrolment of CALD populations in clinical trials.

Trial consents are all in English…You’re supposed to get them…changed into the native language…but that can take a lot of time. There’s strict time requirements for trials…it might take a week, or two weeks to get a trial document transcribed…and in that time their cancer has progressed to the point where they can’t get on the trial anymore.

Interview 14 (Doctor)

When considering the lower enrolment of CALD populations in clinical trials, participants reflected on the role of system constraints influencing clinician decision-making.

That’s well known about CALD patients and trials… and there’s probably a reluctance from clinicians to enrol [CALD] patients on trials because it just becomes more difficult…it’s not really a fair or just thing, but it’s how…in the current system, the way it’s set up.

Interview 14 (Doctor)

This was echoed by two clinical trial coordinators who discussed challenges in enrolling patients who require an interpreter.

So they miss out or are not even put forward…they could benefit…either they would take too long just to complete the eligibility criteria…it takes too long to get things translated…then they might be ineligible…because the screening period has passed.

Interview 16 (Specialist Nurse)

So you make sure you book an interpreter for the entire time, which you get questions about, ‘what do you mean you need it for an hour?…it’s very expensive’…they’ve been pushed back from a financial point of view.

Interview 1 (Specialist Nurse)

Clinicians reflected on instances where an interpreter was not present, such as daily ward rounds. Clinicians reported feelings of unease with these communication practises.

At the bedside, multiple teams, and nurses…just standing in front of them…talking about them and they’re not understanding…we talk really fast…it would just feel like a fly on the wall, just sitting in your bed, looking at everyone around you do this.

Interview 17 (Registered Nurse)

### Use of Multilingual Staff

The use of multilingual staff was presented both as a facilitator and barrier to care provision. Many participants reflected that having a multilingual workforce was very useful, particularly in day-to-day operations and communications. However, participants also acknowledged they “weren’t meant to” use multilingual staff as translators and that they did worry about the burden this practise places on staff. One staff member discussed their experience of being asked to interpret for a patient without any formal training.

I felt I had the responsibility to do, to attend to everything. I could help, but then I also couldn’t… Yeah, I felt a bit pressured…the doctor asked me to give his wife a call…because she doesn’t speak English…when I gave her a call she burst into tears…she knew he was going to surgery but didn’t know what they were doing…

Interview 17 (Registered Nurse)

This participant explained that the responsibility of interpreting added pressure to their usual roles and responsibilities.

I was like, stressing about the shift… I went upstairs to see him…gave his wife a call and then came back down, back to my shift. Yeah, it was an emotional rollercoaster.

Interview 17 (Registered Nurse)

### Use of Family Members

Similarly, participants discussed the usefulness of including family members in patient discussions and utilising them as interpreters in the absence of, or in combination with, trained interpreters. One participant reflected on the moral conflict they experienced when using family members but acknowledged there is sometimes no alternative.

You get them [translators] for 20 minutes a day and the rest of the time you’re drawing pictures… doing charades around end-of-life care. Its woefully inadequate… there are a number of practices… that ethically I feel challenged about…I’ve had to do in the complete absence of any other solution.

Interview 20 (Executive)

These feelings were further explored by this participant.

You know, having to use an 8-year-old to do it…interpretation around death. Definitely not gold standard. The alternative is to leave a family totally isolated…wherever possible you avoid doing things like that…but at times…it’s been necessary. You feel incredibly sad…even thinking about it now…I wonder how those children fared long term. I wonder how families fared long term.

Interview 20 (Executive)

Despite some associated moral conflict, family was largely observed as an enabler for communication.

I try and encourage as many family members as possible to come along. There is a lot of information, and more than one person helps.

Interview 15 (Doctor)

## Structure

### Informal Mechanisms

Participants described a reliance on informal mechanisms for communication as well as information and resource sharing. This included staff being heavily reliant on the information relayed during handover between clinicians, and doctors relying on nursing staff and care coordinators to “flag” individual patient needs. Some staff members felt that when this informal mechanism worked well, multi-disciplinary communication was a facilitator to care. However, many staff members described experiences of poor and ad hoc information sharing, which left staff members feeling unsupported and unprepared to provide quality care.

You receive a patient…they’ll arrive on the ward…not English speaking… it’s not always handed over. Right ok. This is completely not what I expected.

Interview 17 (Registered Nurse)

…there’s a real gap for identifying CALD patients and… for supporting staff to accurately and safely identify. So, when clinicians say we don’t have a process or a way, they’re not completely wrong.

Interview 13 (Executive staff)

### Assessment of Needs

Clinicians reflected on the assessment tools available in their practise and their lack of responsiveness to identifying or meeting CALD patients’ needs.

It’s really hard to say that there’s any mechanism of identifying if were doing that well, or even at all…meeting their [needs], what’s acceptable to them… we probably look at what’s acceptable to us.

Interview 6 (Registered Nurse)

Non-clinical participants reflected on the skills of clinicians, suggesting that it may be a lack of clinical engagement, leading to patient needs not being identified or assessed adequately.

Clinicians hold all the power

Interview 8 (Executive)

### Inflexible System and Clinician Scope

Participants described the structure of the institution as highly inflexible. This was seen as a significant barrier to equitable care provision, as many participants felt this required them to go beyond their scope to meet patient needs. This produced some moral conflict in participants.

Sometimes I find I need to overstep my boundary in my position to be able to provide this patient with additional care…just sort of hoping that they [other staff members] will do the same kind of job that you would, but you don’t actually have a way of knowing.

Interview 1 (Specialist Nurse)

I would say it’s quite frustrating to feel that something needs to be done, but not be able to do it.

Interview 5 (Doctor)

### Training and Education

No participants could identify any specific training they had experienced regarding caring for CALD populations. Many participants reflected on their own work experiences being their education.

Lessons learned; they don’t teach you that in nursing school.

Interview 19 (Specialist Nurse)

That’s been from the school of Hard Knocks, it’s literally being at the coalface, getting confronted by these things.

Interview 8 (Executive)

Some participants described feeling unsupported when put in complex situations, without formal training. One participant described moral distress associated with looking after a complex patient from a refugee background.

Totally, totally unsupported. And I actually found it quite distressing to be honest. And I don’t think there was any real acknowledgment of the fact that this was quite a distressing situation… He had escaped his country… he couldn’t speak English, he had no family and he had been told he was dying… and he was being treated like a criminal, tied to his bed… The whole thing felt so confronting and we didn’t really receive any education or support…

Interview 6 (Registered Nurse)

## Discussion

To our knowledge, this is one of few qualitative studies to comprehensively assess the institutional factors associated with equitable care provision for CALD populations in Australia, and the first in the field of cancer care. Findings demonstrate that despite being a multicultural and multilingual society dedicated to UHC, the Australian healthcare system remains tethered to the historical legacies of colonialism and the White Australia Policy [48, 49]. Although the Australian healthcare system has introduced incremental policy advancements aimed at supporting CALD populations, such as the Queensland Health Multicultural Policy and Action Plan [50], institutional factors continue to influence health inequities [38, 51]. This is evidenced by an inflexible, white-centering health system that privileges the needs of the English-speaking majority and fails to provide the requisite structure and resources to enable the provision of equitable care for racialised or minority populations. This reinforces a deficit perspective of CALD populations, as it strengthens the perception that they are inherently, rather than structurally vulnerable [1, 48, 52]. This was also found to produce moral conflict and distress in healthcare staff, due to a discordance between their professional values and daily practises [53, 54].

A key finding was the strong focus of participants on language needs, with minimal attention given to the cultural identities or needs of patients. Clinicians overwhelmingly identified CALD patients as those with perceived limited English proficiency (LEP). Consequently, they viewed those patients as vulnerable or disadvantaged. Communication was the key barrier to care provision identified throughout the interviews. Staff reported that insufficient or delayed access to interpreter services, results in suboptimal practises, including simplifying or reducing health information, using family members and multilingual staff as informal interpreters, reduced clinical trial information and enrolment, and the avoidance of probing questions, particularly regarding patients’ psychosocial needs. Previous Australian research has demonstrated that patients with LEP often experience modifications to standard care, resulting in poorer quality care [55]. It has also been reported that staff caring for patients with LEP modify their approach by ordering additional clinical tests, using multilingual staff and family as “pseudo-interpreters”, communicating information indirectly, and delivering large amounts of information during infrequent interpreter sessions [51, 56].

Use of terminology such as “language barriers” perpetuates the misconception that communication challenges result from an individual or community deficit, rather than institutional limitations, such as inaccessible interpreter services [57]. The deficit narratives that are furthered by such terminology are incongruent with Australia’s linguistic diversity, where a quarter of Australian households currently speak a language other than English, and proficiency in two, or even three languages is commonplace for many CALD populations [58]. The institutional focus on “language barriers” furthers the hegemony of English-speaking populations in health systems whilst neglecting the critical need for services to adapt to Australia’s evolving multilingual landscape [54, 57]. This deficit terminology was challenged by a CALD-identifying participant who emphasised that LEP does not equate to low health literacy, but rather represents an institutional barrier that reduces access to information. Another participant reflected that these institutional barriers limited those with LEP from self-advocating during substandard care, leading patients to fall “out of the priority list”. Furthermore, the prevailing institutional focus on communication challenges currently overshadows their obligation to address patients’ diverse cultural needs. We suggest that future research utilises strengths-based terminology and explores the integration of both the cultural and linguistic needs of patients within health institutions.

Another key finding was the reliance on assumptions and informal mechanisms for the identification of CALD patients, assessment of their needs, multidisciplinary communication, and resource-sharing. This approach is problematic, as research has demonstrated that implicit and informal mechanisms are inadequate for effective prioritisation of health equity [54, 59]. The absence of formalised mechanisms positions health equity as an implicit, rather than explicit goal within health services and leaves CALD populations vulnerable to individual clinician attitudes and highly variable care, which can be compounded within busy clinical environments [60]. To address this, it is imperative that health equity be embedded within health institutions through the implementation of explicit policies and practises, for example using patient-driven co-design to improve patient registration forms [59, 61].

Embedding equity within health systems has been investigated by van Roode and colleagues, who concluded that equity must be prioritised through both informal and formal mechanisms [54]. Informal mechanisms include institutions adopting equity values and engaging equity champions [54]. Formal mechanisms involve explicit institutional commitments, such as requiring health equity in decision-making, the allocation of appropriate resources, and building capacity and coordination across the health system [54]. A critical need identified in this study is timely access to interpreter and transcription services for clinical trial information and consent forms. This is of particular urgency due to the well-documented underrepresentation of CALD populations in clinical trials [62]. Formal commitments also require the explicit naming of equity goals, with measurable outcomes and targeted solutions [54, 63]. These factors have been substantiated through international health equity actions and implementation frameworks [59, 64–66]. The present study displays that Australian health services are engaging with some informal mechanisms associated with health equity prioritisation, including adopting equity values such as “safe, equitable, and culturally appropriate public healthcare” [50]. However, health equity must also be structurally embedded through formal mechanisms. Central to structurally embedding equity within health systems is co-design and co-production of system policies and practises with CALD community members [67]. This is necessary to address institutional racism and the pervasive deficit discourse in the Australian health system but has also been shown to be a strength-based and solution-focussed approach to system change [68].

A third key finding was the common experience of moral conflict and distress for clinicians, indicating systemic failures in supporting both CALD patients and clinical staff. Moral distress refers to clinicians who are compelled to act in a way that conflicts with their professional values, due to institutional constraints [59, 69, 70]. Previous research has asserted that clinician moral distress is increased within systems that encourage individual responsibility regarding the rationing of healthcare and resources [69]. This is particularly evident amongst populations who may be structurally vulnerable, such as CALD populations, as it causes clinicians to prioritise or deprioritise care according to available resources, as displayed in this, and other studies, by staff reluctance to enrol CALD patients in clinical trials [69, 71]. Previous research has displayed those situations which produce moral distress in healthcare workers often necessitate “hidden work” by clinicians to manage healthcare access gaps [69, 72, 73]. This “hidden work” was particularly evident in this study when considering the additional burden placed on multilingual healthcare staff. It was also evident amongst clinicians who expressed that they felt the needed to step outside the scope of their role in order to provide equitable care. Utilising staff members to compensate for service gaps has long been linked to reduced patient safety and higher rates of clinician burnout [74]. This is a pertinent consideration in the COVID-19 era, which reports high healthcare worker stress, reduced job satisfaction, and increased intentions to leave the healthcare workforce globally [75–78]. Institutional strategies to mitigate moral distress amongst clinicians includes proactive debriefing [79], peer support [80], and policy changes that prioritise staff wellbeing, such as adequate staffing, resources, and access to organisational support [81].

Therefore, this study has demonstrated that without key cultural and structural reform, Australian health systems will remain tethered to their colonial and migratory history and continue reproducing health inequities amongst racialised populations. This cycle can be disrupted through embedding equity within institutional structures [54]. This will enable health institutions to better support CALD patients and the staff providing their care, whilst fulfilling their commitments to providing equitable, high quality healthcare [50, 82].

## Limitations

Whilst this study encompassed a diverse range of health professionals, including some who identified as CALD, there are inherent constraints on the extent to which the participants can provide valid and unbiased interpretations of processes such as racialisation and institutional racism [83, 84]. This arises from their positionality as insiders within the health institution, which may “blind” them to key experiences or perspectives of some patients [34]. Consequently, these findings convey the barriers experienced or perceived by staff within their role providing equitable care. Furthermore, future research would be enhanced by adopting an intersectional approach that examines how various structural determinants, such as socioeconomic status, migration history, gender, and cultural norms interact with healthcare institutions. This approach would recognise the heterogeneity of CALD populations across Queensland and identify their unique needs. Future research should centre the varied perspectives and experiences of CALD patients when navigating the Australian healthcare system. The triangulation of CALD perspectives with hospital policies and guidelines would provide deeper insights into the institutional factors influencing CALD cancer inequities.

## Conclusions

This research has displayed that there is a critical need for health system reform to enable the provision of equitable cancer care. This requires that equity be explicitly promoted through both formal and informal mechanisms. Adapting services to reflect the needs of the current Australian population will benefit CALD, and other racialised or minority populations, as well as reduce moral distress and burnout amongst healthcare staff. This is a necessary step for Australian institutions to begin deconstructing the enduring structures of our colonial history and to promote equitable healthcare for all Australian’s.

## Data Availability

The datasets generated and/or analysed during the current study are not publicly available due to confidentiality concerns and potential identification of interview participants.

## Abbreviations

CALD: Culturally and Linguistically Diverse
OECD: Organisation for Economic Co-operation and Development
UHC: Universal Health Coverage
CRT: Critical Race Theory
LEP: Limited English proficiency
